# A Design-Led Theory of Change for a Mobile Game App (Go Nisha Go) for Adolescent Girls in India: Multimix Methodology Study

**DOI:** 10.2196/43085

**Published:** 2023-04-18

**Authors:** Lalita Shankar, Anvita Dixit, Susan Howard

**Affiliations:** 1 Howard Delafield International LLP Washington, DC United States; 2 Interdisciplinary School of Health Sciences Faculty of Health Sciences University of Ottawa Ottawa, ON Canada; 3 School of Integrative Studies College of Humanities and Social Studies George Mason University Fairfax, VA United States

**Keywords:** Theory of Change, social behavior change, mobile game app, digital innovation, adolescent girls, sexual and reproductive health, India

## Abstract

**Background:**

India has one of the largest adolescent populations in the world. Yet adolescents, particularly adolescent girls, have limited access to correct sexual and reproductive health information and services. The context in which adolescent girls live is one of gender inequity where they contend with early marriage and early pregnancy and have few opportunities for quality education and labor force participation. The digital revolution has expanded the penetration of mobile phones across India, increasingly being used by adolescent girls. Health interventions are also moving onto digital platforms. Evidence has shown that applications of game elements and game-based learning can be powerful tools in behavior change and health interventions. This provides a unique opportunity, particularly for the private sector, to reach and empower adolescent girls directly with information, products, and services in a private and fun manner.

**Objective:**

The objective of this paper is to describe how a design-led Theory of Change (ToC) was formulated for a mobile game app that is not only underpinned by theories of various behavior change models but also identifies variables and triggers for in-game behavioral intentions that can be tracked and measured within the game and validated through a rigorous post-gameplay outcome evaluation.

**Methods:**

We describe the use of a multimix methodology to formulate a ToC informing behavioral frameworks and co-design approaches in our proof-of-concept product development journey. This process created a statement of hypothesis and “pathways to impact” with a continuous, cumulative, and iterative design process that included key stakeholders in the production of a smartphone app. With theoretical underpinnings of social behavior and modeling frameworks, systematic research, and other creative methods, we developed a design-led ToC pathway that can delineate complex and multidisciplinary outputs for measuring impact.

**Results:**

The statement of hypothesis that emerged posits that “If girls virtually experience the outcomes of choices that they make for their avatar in the mobile game, then they can make informed decisions that direct the course of their own life.” Four learning pathways (DISCOVER, PLAY, DECIDE, and ACT) are scaffolded on 3 pillars of evidence, engagement, and evaluation to support the ToC-led framework. It informs decision-making and life outcomes through game-based objectives and in-game triggers that offer direct access to information, products, and services.

**Conclusions:**

This approach of using a multimix methodology for identifying varied and multidisciplinary pathways to change is of particular interest to measuring the impact of innovations, especially digital products, that do not necessarily conform with traditional behavioral change models or standard co-design approaches. We also explain the benefits of using iterative and cumulative inputs to integrate ongoing user feedback, while identifying pathways to various impacts, and not limiting it to only the design and development phase.

## Introduction

### Background

India has one of the largest adolescent populations (253 million) in the world [1], representing a demographic challenge and an opportunity for its fast-growing economy. Gendered expectations for adolescent girls remain key barriers to their health, education, and labor force participation. Studies show that 27% of girls are married before the legal age of 18 and 8% of girls aged 15-19 years have begun childbearing [2]. Several programs within health systems have been improved but remain accessible only to older adolescents [3-5]. Other gaps in knowledge include a lack of comprehensive sex education that would equip adolescents with age-appropriate and scientific reproductive health information [6].

Current programs and policies remain disjointed with gaps in implementation that show a lack of adolescent-responsive services [7]. Health workers and parents continue to be the gatekeepers of knowledge, thus limiting direct access to information and services for adolescents. Increasing young women’s education, workforce participation, and financial independence requires building their knowledge, agency, and ability to avoid such disruptions on their pathway to prosperity [8]. The need for scalable, sustainable approaches for the provision of accurate health information and support for healthy behaviors among adolescents (aged 15-19 years) in India continues to be a challenge.

Engaging with the private sector can play a critical role in leveraging a wide range of resources that can help reach adolescents more directly and at scale [9]. The private sector efficiently supplies products, services, and communications through mass media [10]. Apart from medical or frontline workers, there are few sources of credible information that are available directly to adolescents, who also value privacy and anonymity. Only 20.8% of girls aged 15-19 years report any contact with health care workers [11]. Evidence suggests that many private sector actors value the chance to connect with the adolescent market when they can do so in a way that reduces their reputational risk [12,13]. Using a direct-to-consumer (DTC) approach overcomes these limitations, fulfilling the needs of adolescents and contributing to national goals for adolescent health and development.

### Leveraging the Digital Revolution for Improving Reproductive Health Outcomes Among Adolescents

There has been a significant increase in ownership and access to mobile phones in India, with approximately 62% of urban women owning a mobile phone [2]. There are approximately 1.1 billion mobile connections in India as of 2021 [14]. High mobile penetration has been leveraged by behavior change interventions for improving health and financial autonomy by delivering information through SMS text messaging, smart apps, telemedicine, mobile health solutions, web-based education, and so on [15,16]. COVID-19 has driven up the use of mobile phones by girls in India [17]. Girls describe phones as broadening horizons and providing a gateway to new opportunities [18]. Delivering information and services in a private and fun intervention using mobile games provides a unique opportunity to reach adolescent girls directly and empowers them to become active decision makers in their own lives. Game-based learning can improve health outcomes for adolescents outside the virtual environment [19-21]. Evidence suggests that simulations of behavioral outcomes could impact behavior change by applying knowledge and practice making choices, thus experiencing outcomes of choices through relatable avatars in a game [22].

### Go Nisha Go—A Mobile Game App That Uses a DTC Platform to Reach Adolescents in India

Go Nisha Go is the first of a suite of games under the aegis of the Game of Choice, Not Chance (GOC) project implemented by Howard Delafield International, LLP, a US- and India-based behavioral insights firm. The project uses a hybrid of interactive story-based video games, comprehensive education e-learning tools, simulations, and web portals that challenge players to make decisions and choices within the game, based on realistic scenarios. Launched in June 2022 and available for free download on Google Play, Go Nisha Go uses discovery and play to empower players to become active decision makers in their lives, particularly for improving their sexual and reproductive health (SRH). The 5 episodes cover menstrual health management (MHM), negotiation skills, fertility awareness (FA), consent, contraception, and delay of marriage. The core concepts for each episode include negotiation for individual identity and mobility (episode 1); self-care, particularly for MHM (episode 2); consent and negotiation in relationships (episode 3); contraception methods and FA (episode 4); and self-efficacy through confidence building to navigate one’s future (episode 5). Providing a “virtual private space” to explore life choices, the game is designed to advance and support healthy SRH behaviors and improve attitudes, confidence, and decision support for managing self-care and negotiating relationships with parents, peers, teachers, and partners. The episodes, topics, and storylines were informed by formative research, as well as a continuous and iterative co-design process with a cohort of girls aged 15-19 years in India.

### Challenges of Adapting Theoretical Frameworks for the Game World

Identifying theories informing game design for improved SRH outcomes for adolescents via the DTC platform can be challenging, as over 1700 constructs have been identified within 83 health behavior theories [23]. Some of these theories have overlapping constructs [24] that can be operationalized separately [25]. Recently, attempts have been made to combine theories [26], but these efforts are still in their infancy. These collections of widely cited health behavior theories are intended to inform the design and implementation of health care interventions. The United Kingdom Medical Research Council has recognized the importance of complex interventions informed by behavioral theory and has detailed guidance about how to use them [27]. Fogg [28] focuses on behaviors that are entwined with technology to launch the intervention, whereby pervasive technologies are typically intended to support motivation or ability, as well as provide an active trigger for behavior.

Measuring the efficacy of the proof of concept was initially a commitment to funders only through game analytics that would capture incremental changes in knowledge, skills, attitudes, and in-game behaviors. However, there was an acknowledged need to provide more valid assessments beyond game heuristics to support or refute efficacy [29-32]. A meta-analysis of “serious games” to study the effectiveness of interventions for SRH promotion [33] concluded that there was a need for studies with rigorous evaluations of game effectiveness, longer-term follow-up, and using measures of behavior, rather than merely their determinants.

### Objectives

The objective of this paper is to describe a design-led Theory of Change (ToC) formulated for a proof of concept (a mobile game app to improve SRH outcomes in adolescents) that is underpinned by theories of various behavior change models using a multidisciplinary methodology. We also identify impact pathways through an iterative process that can or will be validated through a rigorous gameplay outcome evaluation. We used a design thinking lens for product development, which is a nonlinear, iterative process that facilitates user-centric problem-solving by understanding users, challenging assumptions, redefining problems, and creating innovative solutions to prototype and test.

Our ToC enables the validation of the proof of concept. Multimix methodologies were followed in three distinct phases: (1) formative research, (2) game design and development, and (3) game deployment and evaluation, which contributed to various outputs that eventually identified the discrete measurement pathways for outcomes and impact. This paper describes these processes and multimix methods.

## Methods

### Formative Research

We conducted initial desk and literature reviews and followed up with formative research to gather evidence of current knowledge, attitude, and practices of SRH among adolescent girls, with respect to their sense of identity, role models, family, education, dreams, fears, and decision-making power.

We used semistructured interviews and visual stimuli to elicit insights from girls (aged 15-19 years, N=103). After participants expressed perceptions of social norms, moral standards, obligations, and aspirations, we carried out a thematic analysis using predetermined codes and used inductive analysis to identify emergent themes. The results led to the identification of 4 predominant psychographic personas that emerged as descriptive categories of girls’ lives and attitudes. These 4 profiles informed the scenarios, in-game decisions, and relatable content [34].

### Study of Social Theoretical Frameworks and Models

A review of social theoretical frameworks and models included desk and literature reviews with reference to game-based learning, social theoretical frameworks, behavior change models, technological behavioral intervention models, DTC market approaches, and participatory ToC frameworks for complex interventions. Additionally, one of the authors (SH), the GOC Project Director, also provided evidence from her research on the application of game-based learning in One Health [35] to adapt those techniques to the development of the mobile game app. We conducted additional literature reviews of studies on consumer behavior models and frameworks most relevant to the game, to support the design of seamless DTC in-game elements.

### Co-design Workshops and Consultations

Co-design workshops planned by game designers, data visualizers, and gender researchers were conducted with participants from 3 North Indian cities (12 sessions each with 24 girls, aged 15-19 years, owning smartphones). The goal was to gather tangible, detailed, and qualitative feedback from prospective end users (players) that would directly inform game design and development, effectively engaging the player in a relatable manner. Twelve waves of data collection were carried out, each with its own objective, testing specific components for the game: mini-game concepts, contextual relevance of specific episode narratives, game “win-loss” state and scoring, voiceover artists’ likability, visual references, preferred DTC products and care, and conflicting challenges that girls face and their aspirations. A detailed description of the co-design and design testing study is documented elsewhere (N Mohandas et al, unpublished data) and is not described further in this paper.

### Curating Elements for the DTC Experience

Recognizing the value of engaging the private sector and other organizations with youth-responsive products and services, a partnership strategy was developed using a stakeholder mapping process. We identified product categories, as well as information services, that were appropriate and relevant for a game for adolescent girls aimed to improve agency and SRH outcomes. These categories include products and services, beyond SRH such as helplines, academic and career skill-building tools, and personal development.

### Game Design and Development

The game design and prototype development phase followed iterative “design sprints”—where user engagement is captured to drive game elements. The game mechanics for a DTC platform were designed to optimize decision-making in a fun and educative way. We again used multimix methods to create a choice-based game narrative, creating in-game challenges that addressed complex social norms that adolescent girls face during decision-making, that is, a pull toward following their dreams and aspirations versus a push to conform to expected gender-based stereotypes. Cumulative and iterative inputs from end users were continuously solicited through design testing workshops and rapid prototyping sessions. Thus, we established a relevant user experience and appeal of the game and identified learning objectives and data collection points for a dashboard of indicators to measure in-game metrics.

### Multidisciplinary Consultations for Collaborative Consensus

The team’s weekly game design and development meetings actively engaged a multidisciplinary global team of game designers; product managers; public health and gender experts; researchers; academia; technical advisors in adolescent health; computer engineers; data analysts; visual and communication designers; marketing, communication, operations experts; and funders. Monthly, quarterly, and semiannual meetings with advisory board members, consortium members, funders, and others were also held to review our progress, seek feedback, and improve iterations to game development. This collaborative engagement ensured buy-in and consensus. These processes contributed to the development of the learning objectives for the game, firming game elements and game analytics for the game, development of the partnership strategies for providing in-game resources, development of the marketing strategy for influencing downloads and repeat gameplay, and predictive analytics (PA) modeling incorporated into the game. They also informed the external outcome evaluation study designs and protocols and identified data collection points along the impact pathways.

### Developing a Hypothesis for the Proof of Concept (Game App)

Game learning objectives were determined, followed by game elements, to improve decision-making and agency through scenario-based stories with conflicts and challenges. There are opportunities for discovery through mentors (nonplayer characters) and choice architecture that reflects the 4 personas and the variety of choice options. A three-pronged research approach to determine game impact was also formed: (1) in-game metrics covering levels of incremental knowledge and attitudes for MHM, FA, contraception, consent, and demonstration of confidence in decision-making; (2) data from PA modeling within the game for precision messaging, leading to increased efficacy and impact; and (3) a longitudinal randomized controlled trial (RCT) for external game evaluation to demonstrate impact.

Multiple brainstorming web-based workshops were conducted with the team using the Miro software to outline a storyboard, layering all the data sets, and to define objectives, use cases, and delivery of choice architecture [36]. A participatory method was used to form a hypothesis that best describes the proof of concept and its intended impact. Subsequently, a visual design exercise was conducted to illustrate the hypothesis as part of an overarching, design-led ToC that mapped the problem statement, the various inputs, the multidisciplinary pathways to outcomes, and data collection points for the intended impact.

### Ethical Consideration

No primary studies were carried out; thus, no data sets were generated or analyzed during this study. Standard ethical considerations were followed during research for the previous studies that informed the ToC, including consent from adolescents and parents, when necessary. All data that were used to inform the ToC development were private and confidential as required by the Data Protection and Privacy Act regulations in India. For the formative research, the FHI 360 Office of International Research Ethics determined that further review and approval of design testing project components is not required because the project does not meet the regulatory definition of research as defined under the Department of Health and Human Services Code of Federal Regulations (review ID: 1646613). All authors had completed the Human Subjects Research Collaborative Institutional Training Initiative (CITI Program) certification.

## Results

In developing a proof of concept to demonstrate our hypothesis for improved SRH outcomes that reach end users directly, we identified 4 learning pathways that were scaffolded on the pillars of evidence, engagement, and evaluation.

These pathways, designated as DISCOVER, PLAY, DECIDE, and ACT, were informed by the collective multimix study described above.

We developed the final hypothesis for our proof of concept that posits that “If a girl experiences the simulated outcome of her avatar’s choices on her health, relationships, and confidence, through a game that offers elements of immersive engagement, challenge, and fun, and ‘nudges’ her to access information, relevant products, and appropriate services directly, then the girl will ‘learn’ to make informed decisions about SRH that will positively impact the course of her life.”

In the results shown in Table 1, we highlight how we aligned DISCOVER, PLAY, DECIDE, and ACT to various methods such as formative research, sociobehavioral theories, game-based learning theories, and consumer behavior models to subsequently inform, design, develop, and evaluate our game (product) intervention.

**Table 1:** Social theoretical frameworks and their application to Go Nisha Go.

Social theoretical framework	Description	How we applied it to the game
The Proteus Effect [37,38]	Describes the phenomenon in which the behavior of an individual within a virtual world is changed by the characteristics of their avatar Based on the hypothesis that an individual’s behavior conforms to their digital self-representation independent of how others perceive them	Guided the creation of a relatable avatar Allows players to customize clothing, hairstyle, etc, with choices and options that reflect the personas identified through the formative psychographic research Identified optimal conflict and challenges where the Proteus Effect would be manifested
Prensky’s Framework of Learner Engagement [39,40] Game-based learning and goal setting [20,21,35]	Recognizes that people respond more effectively to speed, fun, and graphics Proposes engagement with fast-paced games with serious content to create more effective learning	Provides a goal that is compelling Engaging through a conflict or challenge Allows for immersion through discovery and play (eg, exploration of towns, mentor or role model characters, customization of clothing, hair, etc) Engages the player in decision-making and shows the outcome of that choice Sounds and visuals that the game offers are further designed to make it compelling
Social Learning Theory [41] Motivation Theory of Role Modeling [42,43]	Postulates that an individual’s cognitive abilities factors, as well as reactions of others within their environment, lead to behavior change through observation, modeling, and imitation Role models can inspire and motivate behavior and goal adoption	Game design actively sought inclusion and design of nonplayer characters in the game who act as role models Nudging players on a journey of social learning and motivation through role modeling Game includes female role models with aspirational careers: scientist, YouTube influencer, nurse entrepreneur, and senior police officer Considerations of diversity were made to ensure increased minority and gender representation of these characters
Self-determination Theory of Motivation [44]	Suggests that people are driven by a need to grow and gain fulfillment People become self-determined when their 3 basic psychological needs of autonomy, competence, and relatedness are fulfilled Offering positive encouragement and feedback boosts intrinsic motivation	Game introduces characters that a player would want to be connected with who are important to them Identifies intrinsic and extrinsic motivation, which leads to determination of 3 “vitals”—health, relationships, and confidence—which represent the trade-offs in choices Provides feedback (magic diary) reflection (mini games) and opportunity to try again (replayability) Allows opportunities to learn skills (prepare a CV, apply for a job, negotiate) In-game links to safety apps, videos, etc External motivators in the links Back-end analytics captures scores and provides feedback to the player on their win-loss states
Yale 4Ps Framework for Behavior Change [42]	A broad strategy for nudging behavior toward desirable outcomes to make healthy choices Supports nudges into 4 broad categories: what choices are offered (Possibility), how choices are made (Process), how choices are communicated (Persuasion), and how intentions are reinforced (Person)	Possibility—Scenarios acting as in-game nudges for improving decision-making: gameplay narrative with conflicts presented in each episode and game scoring system Process—Platform offers nudges for decision-making and learning: choice-based options across 5 game levels, dedicated microsite, direct-to-consumer products, a chatbot with over 400 FAQs^a^, precision messaging from predictive analytics Persuasion—Gameplay elements that promote a strong goal orientation, reflection, and playback: talking diary, win or loss states as trade-offs for decisions made in the game Person—Avatar simulation supporting role-play through character customization and adventure travel

^a^FAQ: frequently asked question.

These theories underpinned the creation of the proof of concept as a hypothesis for improving SRH outcomes that reach adolescents directly. The *Discover* and *Play* pathways are reflected in the design of an engaging, challenging, and entertaining game with the following attributes: incorporation of a relatable character, presence of compelling conflicts, branching within the game narrative based on choices made through players’ decisions, an opportunity for feedback and reflection, a mechanism to try again, simulated outcomes, the experience of win or loss states, achievement of game-based learning objectives, all in a fun manner and measuring incremental changes in knowledge and attitudes within the game. The *Decision-Making* pathway is rooted in social learning and the motivation theory of role modeling, which “nudges” players to make informed choices about their health and relationships and to demonstrate confidence. Finally, the *Act* pathway underpins the Yale 4P Framework for Behavior Change that integrates behavioral economics, psychology, and marketing to enable the players’ direct access to information, products, and services within the game, through strategic partnerships with the private sector. Together, these multilayered pathways create a “learning” experience that engages, entertains, educates, and empowers adolescents. This contrasts with more traditional methods that “tell and test” [22].

We determined that the game would have 5 episodes to cover MHM, negotiation skills, FA, consent, contraception, and delay of marriage. Additionally, the scoring mechanism in the game rested on 3 vitals: Self-worth (awareness)—confidence, individuality, exploration; Relationships (experimentation)—negotiation, decision-making, freedom; and Health (information)—physiology, sustenance, and safety. The narrative includes real conflicts, negotiations with family members, travel and exploration, health and career information, a romantic relationship, self-reflection, and avatar customization.

The specific learning objectives that were defined through the multimix methodology for each of the game levels are outlined in Table 2.

**Figure 1:**
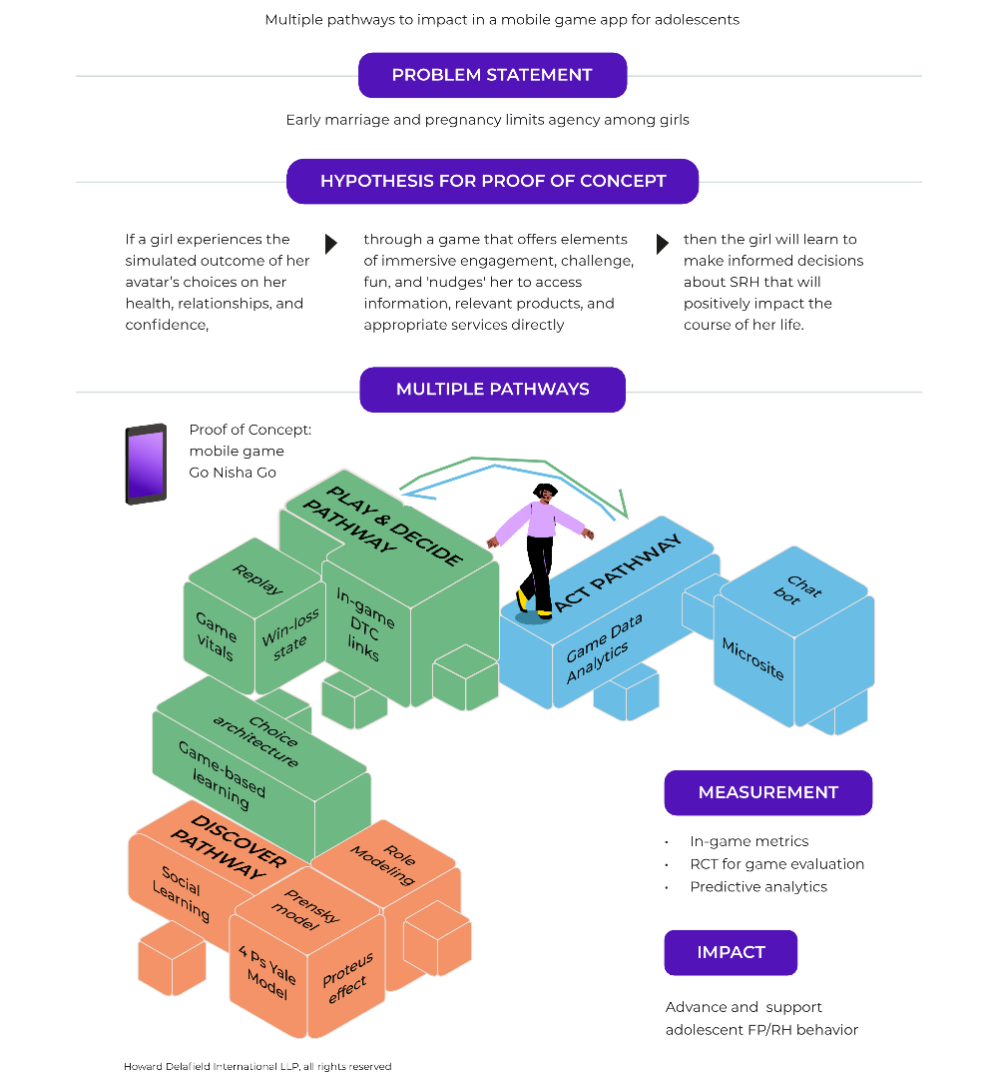
Theory of Change for the Go Nisha Go mobile game app. DTC: direct-to-consumer; FP: family planning; RCT: randomized controlled trial; RH: reproductive health; SRH: sexual and reproductive health.

The visual mapping of the design-led ToC identifies specific data collection points along these learning pathways, which have outlined a four-pronged approach for impact measurement of the learning pathways: (1) in-game metrics for capturing incremental learning across game levels, (2) PA data from machine learning modeling based on choices made by players, (3) in-game digital analytics for the impact of the prototype in the market, and (4) an external RCT-led outcome evaluation, with longitudinal follow-up for reliable measurements of scalable and sustainable impact (Figure 1).

**Table 2:** Learning objectives defined through the multimix methodology for each game level in Go Nisha Go.

Episode	Intermediate result achieved	In-game learning objective
1	Improved linkages to services, products, and information	Ability to access career services tools and information
2	Improved knowledge of and ability to manage menstruation	Ability to persuade or negotiate with family members to participate in normal daily activities while menstruating
3	Improved attitudes, confidence, and decision support for managing her self-care, negotiating consensual sex and contraceptive use, and accessing or using products, information, and care	Ability to evaluate consequences and appropriately explain, persuade, and negotiate when and with whom to have sex, including refusing sex
4	Improved attitudes, confidence, and decision support for managing her self-care, negotiating consensual sex and contraceptive use, and accessing or using products, information, and care	Ability to negotiate contraceptive use with partner
5	Improved assertion of self-identity demonstrated through career, safety, and personal well-being choices Empowered to take accountability for her choices and recognize that their decisions matter	Ability to negotiate partner and career of choice with parents

## Discussion

### Principal Findings

Our ToC approach used a multimix methodology for identifying varied and multidisciplinary pathways to change. The use of an iterative and cumulative process that combines social, behavioral, and game-based learning theories with DTC approaches designed within a mobile game is of particular interest to measuring the impact of innovations, especially digital products. Traditional design and evaluation of health behavior programming implementation does not typically consider the multilayer factors of decision-making that a 3D game can illuminate and simulate. The resultant ToC is design led and serves as a benchmark in the game intervention design and evaluation. The ToC illustration serves as a blueprint for the proof-of-concept hypothesis by defining the functional design, development, deployment, and eventual evaluation metrics for use by a global, multidisciplinary team that comprises technical, technological, and programmatic stakeholders.

In the early stages of game development, the focus of the game evaluation was limited to designing heuristics and in-game dashboards that could measure incremental changes in knowledge through in-game behaviors. However, through the application of a design-led, multilayered approach, the ToC evolved to support key outcome indicators from the game-based learning objectives to include indicators on:

- Improving knowledge about SRH care, understanding the fertile period, and MHM practices.
- Improving attitudes, confidence, and decision support for managing self-care, negotiating consensual sex, contraceptive use, and accessing DTC information, products, and services.
- Improving assertion of self-identity through DTC linkages to career, safety, personal well-being, and improved self-efficacy through direct access to SRH information, products, and services.

Crafting a ToC in the context of an intervention at the crossroads of consumer behavior or human behavior change, technology, game-based learning, and private sector engagement or DTC platforms is complex. Because we conducted an iterative process with multilayer theories and modules, our impact evaluation will be more rigorous. This is because a comprehensive design-led ToC made it easier to identify the data collection points across the product (proof-of-concept prototype) life cycle. This ensured that gold standards of evaluation and validation of the innovation, through an external RCT outcome evaluation to measure larger health outcomes, were complemented by in-game metrics and game heuristics.

Although the iterative and cumulative nature of the ToC is a strength, it was also a challenge to marry science and research with pragmatic product development. We needed to craft a relevant ToC for the proof of concept and measure its impact pathways to validate a complex hypothesis that posits that “If a girl experiences the simulated outcome of her avatar’s choices on her health, relationships, and confidence, through a game that offers elements of immersive engagement, challenge, and fun, and ‘nudges’ her to access information, relevant products, and appropriate services directly, then the girl will ‘learn to’ make informed decisions about SRH that will positively impact the course of her life.” This hypothesis was derived from marrying traditional “old world,” evidence-based, theoretical frameworks guiding game-based learning, and social behavior change communication principles, to create a “novel” intervention (mobile game)—a functional prototype that could facilitate a fun, scalable, and DTC platform to reach adolescents directly with information, products, and services.

The challenge is to ensure that all elements central to the intervention are reflected in the ToC to measure impact only augmented, as game design and development progressed with more elements of game interactivity, and with continuous iterations from the rapid prototyping processes. Additionally, the mobile game generates evidence through in-game, choice-based analytics, embeds interactive content through Chatbots, and has the potential to produce precision messaging and personalized resources through PA and machine learning. The game app is also a data collection tool used by players themselves to improve health outcomes and strengthen personal agency. Understanding the interdependencies and the myriad game elements, including in-game links to products, services, and information on the DTC platform, could affect variables on the pathway to change, a key consideration when designing the game outcome evaluation.

These elements necessitated the development of a robust ToC to create more design and user-centric pathways to describe multiple factors contributing to impact. A 2-arm RCT outcome evaluation, using an encouragement design [45], will be implemented in late 2022 to evaluate the game intervention. We anticipate stronger evidence for the scale-up of the game in addressing SRH and empowerment outcomes beyond the current game. The results of the outcome evaluation will be published as a separate series, with an exclusive focus on the study design, research methodology, and results of the longitudinal study.

### Conclusions

We recognize that a direct-to-adolescent approach, with little or no opportunity to engage parents, teachers, or health providers, could be a radical intervention to increase reach among adolescents who value privacy and anonymity. Research is needed to determine whether these benefits outweigh traditional interventions, which involve gatekeepers of SRH information, products, and services.

The unique, design-led ToC framework is iterative and tailored for the mobile game app, which marries a problem-driven, behavioral science perspective with functional product or prototype metrics. The ToC tests the proof of concept as a hypothesis, which is derived from multiple theoretical approaches and empirical evidence that guided the design and development of the game. The focus, however, is on the end user, the adolescent, which is critical for studying the impact of multiple factors across disciplines governing the intervention. This ToC framework is not derived from 1 theoretical model but arranges disparate theories governing different elements of the proof of concept, systematically and coherently outlining multilevel impact touch points within the game, which could be data collection points for game evaluation. The multilayered, complex intervention for the validation of the proof of concept and its overarching ToC is easily depicted to illustrate 4 central elements of a player’s ability to Discover, Play, Decide, and Act, which are scaffolded over 3 guiding principles of Evidence (theoretical models and formative research), Engagement (in-game mechanics, gameplay elements, and DTC in-game partnership links to products, services, and information), and Evaluation (steering the hypothesis to an impact causal pathway for validation and scale). When there are multiple pathways of outcomes leading to impact, marrying a traditional ToC to a tailored digital impact pathway can support the evaluation of complex interventions with multiple impact touch points. In the game, these include the DTC approach, providing easy, private access to links for SRH information, products, and services, made possible through strategic partnerships with the private sector. We also use in-game metrics and win or loss states determined by choices underpinning relationships, health, and confidence among girls through scoring. The Go Nisha Go game was launched on Google Play in June 2022. The combined ToC and design-led framework paves the way for robust validation of a proof-of-concept prototype, designed to identify potential outcomes and data collection points that address multiple impact pathways.

## Abbreviations

DTC: direct-to-consumer
FA: fertility awareness
GOC: Game of Choice, Not Chance
PA: predictive analytics
RCT: randomized controlled trial
SRH: sexual and reproductive health
ToC: Theory of Change

## Data Availability

Data sharing is not applicable to this paper as no data sets were generated or analyzed during this study.
